# Generation of ventilation/perfusion ratio map in surgical patients by dual-energy CT after xenon inhalation and intravenous contrast media

**DOI:** 10.1186/s13019-018-0737-2

**Published:** 2018-05-18

**Authors:** Kohei Aoki, Yotaro Izumi, Wataru Watanabe, Yuji Shimizu, Hisato Osada, Norinari Honda, Toshihide Itoh, Mitsuo Nakayama

**Affiliations:** 1 Department of General Thoracic Surgery, Saitama Medical University, Medical Center, 1981 Kamoda, Kawagoeshi, Saitama 350-8550 Japan; 2 Department of Radiology, Saitama Medical University, Medical Center, 1981 Kamoda, Kawagoeshi, Saitama 350-8550 Japan; 3 Research and Collaboration Department, Siemens Healthcare, 1-11-1 Osaki, Shinagawa-ku, Tokyo, 141-8644 Japan

**Keywords:** Dual energy CT, Xenon CT, Perfusion CT, Ventilation perfusion ratio

## Abstract

**Background:**

While many studies have evaluated the change in lung volume before and after lung resection and correlated this with pulmonary function test results, there is very little evidence on the changes in ventilation perfusion ratio (V/Q) before versus after lung resection. In the present pilot study, we evaluated if V/Q mapping can be constructed using dual energy CT images.

**Methods:**

Thirty-one lung cancer patients planned for pulmonary resection were included in this study. To evaluate ventilation, Xenon-enhanced CT was performed. This was immediately followed by perfusion CT. The two images were registered manually as well as using dedicated softwares, and division between ventilation pixels and perfusion pixels were done to produce the V/Q map. Also, in order to characterize the distribution of the V/Q, the following numerical indices were calculated; mean, median, mode, standard deviation (SD), coefficient of variation (CV), skewness, kurtosis, and fractal dimension (FD). Pulmonary function tests and blood gas parameters were measured using standard institutional procedures.

**Results:**

In the whole group, VC, %VC, and FEV1 decreased significantly after resection. FEV1.0% was increased significantly after resection. No significant changes were seen in PaO2, PaCO2, and DLCO/VA before and after resection. The mean, median, mode, SD, skewness, kurtosis and FD of the V/Q did not change significantly before and after resection. A marginal but significant decrease in CV was seen before versus after resection.

**Conclusions:**

Overall, it was considered that the V/Q maps could be adequately generated in this study. With further accumulation of data, V/Q map generated by dual energy CT may become one of the potentially useful tools for functional lung imaging.

**Trial registration:**

This trial was registered in University Medical Information Network in Japan (UMIN000010023) on 13Feb2013.

## Background

Lung cancer provides one of the most frequent indications for resection of the lung parenchyma. Candidate patients for surgical resection need to be evaluated whether they have adequate pulmonary functional reserve to undergo resection. Pulmonary function test (PFT) using spirometry is the current standard method of evaluating pulmonary function. While many studies have evaluated the change in lung volume before and after lung resection and correlated this with pulmonary function test results [1–3], there is very little evidence on the changes in ventilation perfusion ratio (V/Q) before and after lung resection. This is considered to be at least in part due to the practical difficulty of frequently performing ventilation perfusion scans using radioneuclides, as well as its low spatial resolution. However, since V/Q is a global index of blood oxygenation of the lung, this ratio may be a good numerical indicator to assess the severity of pulmonary functional derrangement.

Nonradioactive xenon gas has been used to assess cerebral blood flow on conventional CT [4] and has recently been proposed as a contrast medium for the diagnosis of lung diseases due to its high density and higher attenuation of X-rays than air. We have recently used xenon enhanced dual-energy CT with a single-breath-hold technique to assess pulmonary ventilation [5]. Using this method, we were able to predict residual pulmonary function after lung resection for lung cancer [6]. Furthermore we have correlated these results with pulmonary function tests and also studied its application for the detection of response to therapeutic agents for chronic obstructive pulmonary disease [7]. Also, dual-energy CT allows extraction of the iodine component from the enhanced lung parenchyma according to the material decomposition theory [3]. This enables three dimensional acquisition of the pulmonary perfusion.

Combining these 2 techniques, we expected to be able to generate a V/Q map. In the present pilot study, we evaluated if this mapping can be constructed using dual energy CT images. We also evaluated the changes in the mapping before and after lung resection. Additionally we tested to see whether certain statistical indices could be applied to quantitatively evaluate the changes in the distribution of V/Q.

## Methods

### Patients

Between March 2013 to August 2015, thirty-one lung cancer patients planned for pulmonary resection were included in this study. All of these patients were diagnosed or strongly suggestive of lung malignancy and judged that surgical resection was appropriate therapy after routine clinical staging procedures. The study was approved by the Saitama Medical Center institutional review board (approval number 700), and registered to the University Medical Information Network in Japan (UMIN000010023). Written informed consent was obtained from all patients.

### Respiratory function evaluations

PFT was done according to institutional practice before surgery and approximately 6 months after surgery. It consisted of measurements of vital capacity (VC), forced vital capacity (FVC), forced expiratory volume in 1 s (FEV_1_), and the diffusing capacity divided by the alveolar volume (DL_CO_/V_A_). All tests were done in the seated position using a dry rolling-seal spirometer (CHESTAC-9800; Chest, Tokyo, Japan). Arterial blood gases and SpO2 were also analyzed before surgery and approximately 6 months after surgery.

### Ventilation CT

Xenon-enhanced CT was performed before and approximately 6 months after surgical resection. We followed our standard scanning method [7]. Xenon is a stable, radiopaque noble gas that occurs naturally in the atmosphere. Its atomic number is similar to that of iodine, and therefore its distribution and volume can be differentiated from lung tissue and calculated based on dual CT imaging. Briefly, patients in the supine position were scanned in dual-energy mode during breath-hold after a single vital-capacity (from maximum expiration to maximum inspiration) inhalation of a mixture of xenon and oxygen in a ratio of 35:65 using a dual-source CT scanner (SOMATOM Definition Flash; Siemens Healthcare, Forchheim, Germany). Scanning conditions were as follows: tube voltages, 140 kV filtered with 0.4-mm-thick tin and 80 kV; beam collimation, 0.6 mm for each of 128 rows; pitch, 0.55; reconstruction section thickness, 1.5 mm; section interval, 1 mm; and reconstruction kernel, D30f medium smooth. The 35% xenon gas was prepared and stored in a xenon gas supplier (Az-726VXetron VI; Anzai Medical Corporation, Tokyo, Japan). The effective radiation dose was estimated from the dose-length product automatically enumerated by the CT scanner and a conversion factor of 0.018 [8]. Sets of xenon-enhanced images and standard lung CT images were generated by 3-material decomposition on a dedicated workstation (Syngo MMWP; Siemens Healthcare, Forchheim, Germany). Parameters of the 3-material decomposition were set to the default of the manufacturer.

### Perfusion CT

Immediately after the Xenon-enhanced CT, perfusion CT was performed. First a test in injection was done under single breathhold same as the xenon-enhanced CT to determine the time density curve of pulmonary artery and left atrium. This was immediately analyzed to optimize the injection and imaging timing to depict the pulmonary artery effectively. A total of 25 mL of iodinated contrast agent (Ultravist, 370 mgI/mL; Schering) was injected intravenously using a dual-head power injector via a vein of the right arm and at a rate of 5 mL/s, followed by a saline chaser of 25 mL at the same injection rate. Image acquisition was done similar to the ventilation CT. Perfusion images were generated similarly as ventilation CT on the same dedicated workstation with a different application (“Perfused blood volume”) with the default parameter settings.

### Generation of V/Q map

**Fig. 1:**
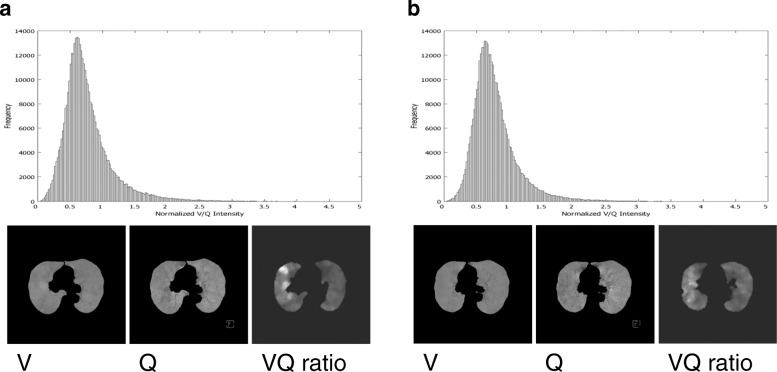
A representative figure of the V/Q overlay and the histogram distribution change are shown before (**a**) and after resection (**b**). This patient developed pneumonia after resection after resection. The V/Q indices before and after resection were as follows; Mean(0.76,0.78), SD(0.38,0.34), Median(0.68,0.71), Mode(0.62,0.62), Skewness(0.02, 0.019), Kurtosis(0.07, 0.07), Fractal Dimension (2.49, 2.49), CV(0.50, 0.44)

Generation of the V/Q map was done as follows. First, linear registration (including no warping) between ventilation images and perfusion images were done manually using contour of the lung as references. Then resampling was done to adjust the slice positions and slice interval using a dedicated software (AZE Virtual Place Plus; AZE Corporation, Tokyo, Japan). Finally, pixel values were normalized for each of the ventilation CT and perfusion CT by the sum of all pixels within the whole lung volume of interest. Division between ventilation pixels and perfusion pixels were done by a dedicated software developed by one of the authors (TI). In order to avoid division by 0, pixels with its value = 0 were replaced by 0 (zero), and pixels with its value =0 in perfusion images was set to 0 (zero) in V/Q ratio map. In this way, normalized V/Q were calculated and mapped. Finally, smoothing was done and the V/Q map was converted to 128 × 128 matrix with application of median filter. A representative figure of the V/Q overlay and the histogram distribution are shown in Fig. 1.

Also, in order to quantitatively characterize the distribution of the V/Q, the following numerical indices; mean, median, mode, standard deviation (SD), coefficient of variation (CV), skewness, kurtosis, and fractal dimension (FD) were calculated. These indices were calculated using software included in the dedicated V/Q software according to standard formulae. FD was also calculated using this software by box counting method as previously described [9]. Only pixels within the lung contours with their values not equal to 0 were used for the calculation.

### Statistics

Data are expressed as mean ± standard deviation. Statistical comparisons were done using paired t-test. Statistical significance was assumed at *p* < 0.05.

## Results

Total of 31 patients were included with mean age of 67 ± 11, ranging from 38 to 86 years old. Male to female ratio was 16 to 15. The surgical procedure was lobectomy in 27 patients, bilobectomy in 1 patient, and pneumonectomy in 3 patients. Surgery was done thoracoscopically in 18 patients, and under thoracotomy in 13 patients.

**Fig. 2:**
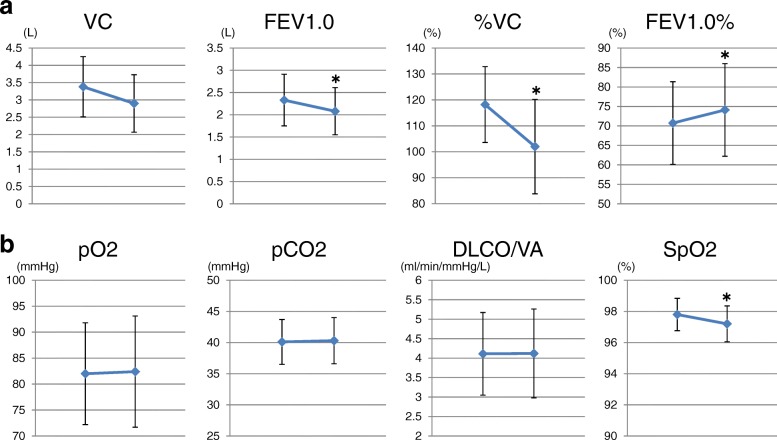
Changes in respiratory functions and blood gas parameters before and after resection. **a** In the whole group, VC, %VC, and FEV1 decreased significantly after resection. FEV1.0% was increased significantly after resection. **b** No significant changes were seen in PaO2, PaCO2, and DLCO/VA before and after resection. SpO2 was decreased significantly after resection but stayed within the normal range. * *p* < 0.05 vs preoperative values

In the whole group, VC, %VC, and FEV1 decreased significantly after resection. FEV1.0% was increased significantly after resection (Fig. 2a). No significant changes were seen in PaO2, PaCO2, and DLCO/VA before and after resection. SpO2 was decreased significantly after resection but stayed within the normal range (Fig. 2a).

**Fig. 3:**
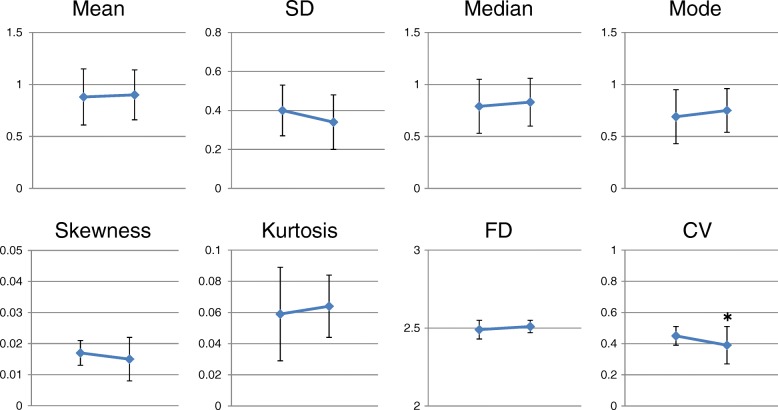
Changes in distribution indices before and after resection. In the whole group, the mean, median, mode, standard deviation (SD), skewness, kurtosis and fractal dimension (FD) of the V/Q did not change significantly before and after resection. A marginal but significant decrease in coefficient of variation (CV) was seen before and after resection. * *p* < 0.05 vs preoperative values

In the whole group, the mean, median, mode, SD, skewness, kurtosis and FD of the V/Q did not change significantly before and after resection. A marginal but significant decrease in CV was seen before and after resection (Fig. 3).

**Fig. 4:**
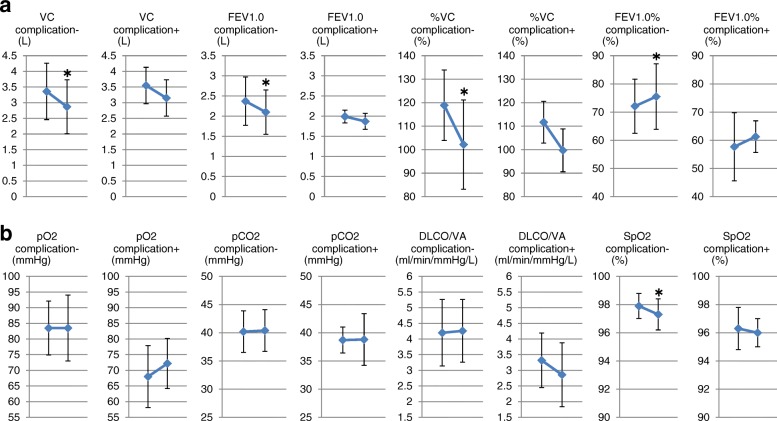
Changes in respiratory functions and blood gas parameters before and after resection in patients with or without post-surgical complications. **a** Pulmonary post-surgical complications were seen in 3 patients with lobectomies. These were acute exacerbation of interstitial pneumonia, pneumonia, and prolonged pleural fistula, respectively. In the whole group, in the patients with versus without complications, the changes in respiratory function before and after resection had similar trends. **b** This trend was also maintained regarding blood gas exchange parameters, except that DLCO/VA was significantly decreased in the patients with complications. * *p* < 0.05 vs preoperative values

**Fig. 5:**
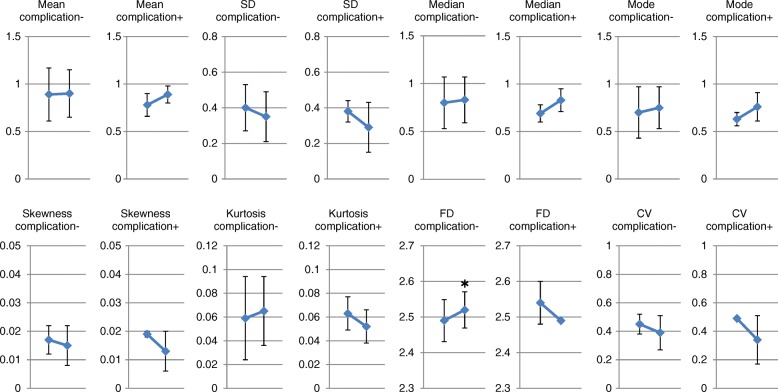
Changes in distribution indices before and after resection in patients with or without post-surgical complications. The changes of the distribution indices of V/Q before and after resection had similar trends except for fractal dimension (FD), which was significantly increased after resection in the group without complications, but had a tendency to decrease in the group with complications. * *p* < 0.05 vs preoperative values

Pulmonary post-surgical complications were seen in 3 patients with lobectomies. These were acute exacerbation of interstitial pneumonia, pneumonia, and prolonged pleural fistula, respectively. In the whole group, in the patients with versus without complications, the changes in respiratory function before and after resection had similar trends (Fig. 4a). This trend was also maintained regarding blood gas exchange parameters, except that DLCO/VA was tended to be decreased in the patients with complications (Fig. 4b). The changes of the distribution indices of V/Q before and after resection had similar trends except for FD, which was significantly increased after resection in the group without complications, but had a tendency to decrease in the group with complications (Fig. 5).

**Fig. 6:**
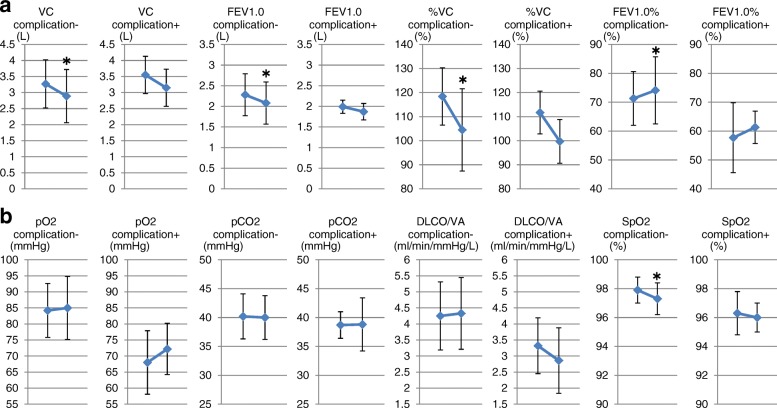
Changes in respiratory functions and blood gas parameters before and after resection in lobectomied patients with or without post-surgical complications. **a** In the lobectomied patients with versus without complications, the changes in respiratory function before and after resection had similar trends compared with the whole group. **b** This trend was also maintained regarding blood gas exchange parameters, except that DLCO/VA was significantly decreased in the patients with complications similar to the whole group. * *p* < 0.05 vs preoperative values

**Fig. 7:**
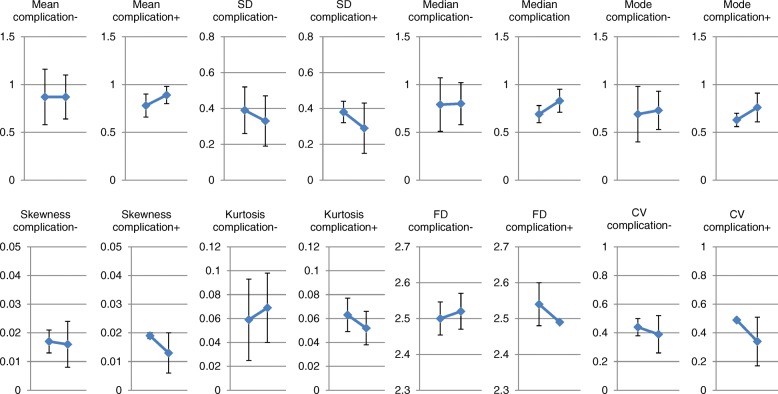
Changes in distribution indices before and after resection in lobectomied patients with or without post-surgical complications. Regarding the distribution indices of V/Q, the changes before and after resection had similar trends with no significant changes in the lobectomy patients

In the lobectomied patients with versus without complications, the changes in respiratory function before and after resection had similar trends compared with the whole group (Fig. 6a). This trend was also maintained regarding blood gas exchange parameters, except that DLCO/VA was tended to be decreased in the patients with complications similar to the whole group (Fig. 6b). Regarding the distribution indices of V/Q, the changes before and after resection had similar trends with no significant changes in the lobectomy patients (Fig. 7).

In the whole group we also compared age of 65 years ≤ versus < 65. The changes in respiratory function before and after resection had similar trends. This trend was also maintained regarding blood gas exchange parameters. Regarding the distribution indices of V/Q, the changes before and after resection had similar trends (data not shown).

**Fig. 8:**
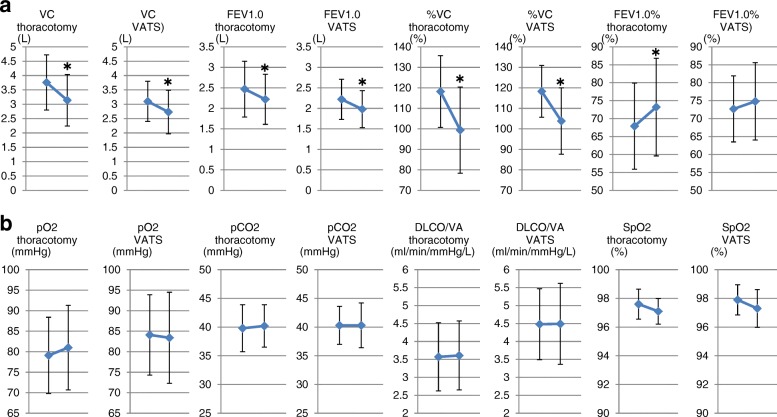
Changes in respiratory functions and blood gas parameters before and after resection in patients with thoracoscopic versus thoracotomy resection. **a** In the whole group we also compared surgical procedure, throracoscopic versus thoracotomy. The changes in respiratory function before and after resection had similar trends. **b** This trend was also maintained regarding blood gas exchange parameters. * *p* < 0.05 vs preoperative values

**Fig. 9:**
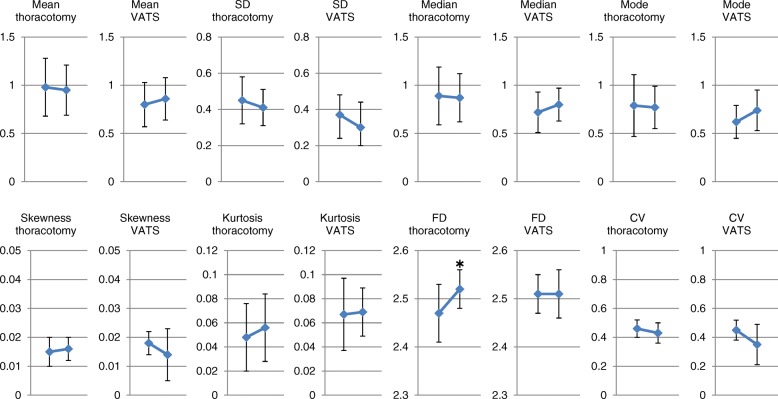
Changes in distribution indices before and after resection in patients with thoracoscopic versus thoracotomy resection. Regarding the distribution indices of V/Q, the changes before and after resection had similar trends except for FD, which was significantly increased after resection in the thoracotomy group. * *p* < 0.05 vs preoperative values

In the whole group we also compared surgical procedure, throracoscopic versus thoracotomy. The changes in respiratory function before and after resection had similar trends (Fig. 8a). This trend was also maintained regarding blood gas exchange parameters (Fig. 8b). Regarding the distribution indices of V/Q, the changes before and after resection had similar trends except for FD, which was significantly increased after resection in the thoracotomy group (Fig. 9).

## Discussion

Overall, it was considered that the V/Q maps could be adequately generated in this study.

In the present study, the xenon images and perfusion images were taken consecutively rather than simultaneously, and were not identical images. Therefore, manual, followed by software driven registration was used to align the two images. Theoretically, by using the current image registration technique, the regional ventilation, perfusion, and V/Q of every pixel in the entire lung could be evaluated. Misregistration of manual registration process primarily due to motion and/or differences in the depth of breathing during imaging acquisition is always a possibility, but at least visually there were minimal errors in the current study. Further improvements in the registration software, such as non-linear registration technique [10], may alleviate these issues.

It is well documented that the uneven distribution of alveolar ventilation and pulmonary blood flow is the most important cause of arterial hypoxemia (with or without hypercapnia) [11, 12]. Alveolar ventilation and perfusion can be indirectly evaluated using PFT. However, the PFT is a global measure of all changes in lung function and does not assess regional changes in alveolar ventilation and pulmonary perfusion. To our knowledge, there are a number of reports of V/Q map generation using dual energy CT [13, 14], but it has not been evaluated in patients undergoing lung resection.

In the present study, overall, VC and FEV1 decreased after resection significantly as expected, but no significant changes were observed pre- and post-resection in the other respiratory function parameters that we have measured. Also, the blood gas analysis data, and DLCO/VA data showed no significant changes pre- and post-resection except for decreased tendency of DLCO/VA in the patients with postoperative complications. Although SpO2 was significantly decreased, the significance of this data is not clear in the absence of significant decrease in PaO2. In accordance with these data, V/Q derived from the dual energy CT was basically unchanged before and after resection. This trend was also similar in the numerical indices of distribution that we have looked at, except for the slight but significant increase in FD which was observed in patients without any postoperative complications, and in patients who received thoracotomy. Our previous study suggests increase in FD derived from V/Q ratio is indicative of increase in self-similar structure [9]. Although the structural significance of this finding remains to be elucidated, it may suggest decreased structural heterogeneity, although more patients will be required to further pursue this finding. This result together with the overall decreasing tendencies in SD, CV and skewness suggest that V/Q may have become more uniform by compensatory mechanisms after resection procedures. Whether this means any improvement in lung gas exchange cannot be confirmed, but both the blood gas analysis data and DLCO/VA data were sustained with no significant changes pre and post resection, indirectly supporting this notion. Notwithstanding, no mechanistic insight is available at this time regarding the association of the V/Q distribution indices and blood gas analysis data, and DLCO/VA data.

There are several drawbacks to this study. Since the outcomes of this study cannot be effectively compared to a gold standard, at present we cannot adequately evaluate the efficacy or significance of this study procedure. Also, all the patients in this study were candidates for resection, which meant that although no selection was done, the patients all had relatively well sustained pulmonary function as well as blood gas and DLCO parameters. Therefore it is quite possible that because of this, no prominent changes were observed in the majority of indices we have looked at before and after resection. Additionally, the methodology in the present study would mean that the perfect for match for V/Q would be 1 instead of the physiologically accepted 0.8 [11, 12], which may need to be accounted for in the interpretation of data.

Since the dual energy perfusion image can also be used for preoperative systemic tumor evaluations as well as follow-up evaluation of metastases for chest, abdomen, and pelvis, the V/Q assessment procedure could be more readily performed in lung cancer patients in comparison to radioisotope scans, despite the fact that the ventilation scan will be an additional examination.

There could be a number of future applications such as the formulation of V/Q map of the region of the lung planned for resection, or the planned residual region of the lung. The predictive benefit of this test for lung resection is too premature to show at this time and remains to be seen, but with further accumulation of data, particularly postoperative complication plus versus minus data, we may be able to find a certain quantitative pattern in V/Q indices which may predict postoperative complications with more accuracy than tests such as SPECT. The validity of this technique may be further evaluated in patients with more extensive abnormalities in the lung parenchyma but still necessitating lung resection. However, higher accuracy particularly in image registration may be required to apply this technique to patients with extensive abnormalities in the lung parenchyma such as three dimensional image registration applying the texture analysis technique [15]. With further accumulation of data, V/Q map generated by dual energy CT may become one of the potentially useful tools for functional lung imaging.

## Conclusions

Overall, it was considered that the V/Q maps could be adequately generated in this study. With further accumulation of data, V/Q map generated by dual energy CT may become one of the potentially useful tools for functional lung imaging.
